# Counterfactual fairness: The case study of a food delivery platform’s reputational-ranking algorithm

**DOI:** 10.3389/fpsyg.2022.1015100

**Published:** 2022-10-26

**Authors:** Marco Piccininni

**Affiliations:** Institute of Public Health, Charité – Universitätsmedizin Berlin, Berlin, Germany

**Keywords:** algorithmic fairness, food delivery platform, counterfactual fairness, reputational-ranking algorithm, causal diagrams

## Abstract

Data-driven algorithms are currently deployed in several fields, leading to a rapid increase in the importance algorithms have in decision-making processes. Over the last years, several instances of discrimination by algorithms were observed. A new branch of research emerged to examine the concept of “algorithmic fairness.” No consensus currently exists on a single operationalization of fairness, although causal-based definitions are arguably more aligned with the human conception of fairness. The aim of this article is to investigate the degree of this alignment in a case study inspired by a recent ruling of an Italian court on the reputational-ranking algorithm used by a food delivery platform. I relied on the documentation of the legal dispute to discuss the applicability, intuitiveness and appropriateness of causal models in evaluating fairness, with a specific focus on a causal-based fairness definition called “counterfactual fairness.” I first describe the details of the dispute and the arguments presented to the court, as well as the court’s final decision, to establish the context of the case study. Then, I translate the dispute into a formal simplified problem using a causal diagram, which represents the main aspects of the data generation process in the case study. I identify the criteria used by the court in ruling that the algorithm was unfair and compare them with the counterfactual fairness definition. The definition of counterfactual fairness was found to be well aligned with the human conception of fairness in this case study, using the court order rationale as a gold standard.

## Introduction

Data-driven algorithms are currently deployed in an increasing number of fields, leading to a rapid increase in the importance algorithms have in decision-making processes (Mehrabi et al., 2019). As their ever-expanding application continues to grow, concerns that algorithms may demonstrate discriminatory behaviors have been raised. In recent years, several cases of biases and discrimination by algorithms were observed (Dastin, 2018; Mehrabi et al., 2019; Obermeyer et al., 2019).

Despite being “data-driven,” algorithms rely on precise development choices and modelling assumptions that inherently reflect specific world views. Moreover, algorithms are trained using historical data that present patterns of association reflecting discrimination and prejudice present in the real world (Kusner et al., 2017; Mehrabi et al., 2019; Wang et al., 2019). Algorithms thus have the potential to introduce and reproduce biased decision-making mechanisms by discriminating against certain individuals (Loftus et al., 2018). For this reason, a new branch of research emerged in the last years focusing on the concept of “algorithmic fairness” (Loftus et al., 2018; Kusner and Loftus, 2020).

A typical fairness problem is that algorithms may discriminate against individuals based on “protected” (or “sensitive”) characteristics such as race, religion, gender, nationality, etc. (Kilbertus et al., 2017; Loftus et al., 2018; Nabi and Shpitser, 2018). Algorithmic fairness researchers aim to understand algorithms’ biases and to impose decision-constraints in order to ensure that such biases are avoided (Loftus et al., 2018; Zhang and Bareinboim, 2018).

A necessary step in achieving this objective is to operationalize the concept of fairness.

## Definition(s) of fairness

Several measures of fairness have been developed. One popular measure of fairness is the “equalized odds” (Hardt et al., 2016). This fairness metric considers an algorithm to be fair if, given the true state of the outcome variable (the variable we are interested in predicting), the predictions are independent of the protected characteristic (Hardt et al., 2016). Another metric is the so-called “calibration.” In contrast, this metric judges an algorithm to be fair if, given the classification of the algorithm, the probability of actually having the outcome variable’s value equal to that of the algorithm’s classification is the same across all values of the protected attribute (Chouldechova, 2017).

Equalized odds and calibration attained notoriety as fairness metrics after both were used to assess the fairness of the COMPAS score (Mitchell et al., 2021). This score was used to predict the probability of a prisoner to commit another crime after release, and the two metrics showed conflicting results in assessing its fairness (Mitchell et al., 2021). Indeed, it was shown that an algorithm cannot generally satisfy these two metrics at the same time (Kleinberg et al., 2016; Loftus et al., 2018).

A further metric frequently used to assess fairness in the machine learning literature is “demographic parity” [or “statistical parity” (Verma and Rubin, 2018)]. Here, an algorithm is considered fair if the probabilities of obtaining a certain class from the algorithm are the same for the different groups determined by the protected variable.

All three of the aforementioned metrics rely on statistical associations between the variables. Several others definition of fairness exists; and other texts provide reviews of the most common definitions (Verma and Rubin, 2018; Mehrabi et al., 2019).

More recently, fairness definitions relying on causal knowledge have gained traction (Makhlouf et al., 2020). Such causal-based definitions are not purely based on statistical associations but rely heavily on external knowledge about real-world processes (Makhlouf et al., 2020). Causal models are difficult to build because a thorough understanding of the context to which the algorithm is applied is required. Nevertheless, they represent a powerful tool to investigate the concept of fairness in decision-making processes (Kilbertus et al., 2017; Loftus et al., 2018; Kusner and Loftus, 2020).

Among the most widely used definitions (Mehrabi et al., 2019) of fairness lies the causal-based “counterfactual fairness” (Kusner et al., 2017). This definition relies on the intuition that fairness can be conceptualized as a thought experiment comparing different scenarios, in which all things are equal aside from forcing the protected characteristic to a different value (counterfactuals) (Loftus et al., 2018).

Despite current lack of consensus on a single measure (Kilbertus et al., 2017), causal-based definitions are thought to be more closely aligned with the human conception of fairness and understanding of discrimination (Pearl and Mackenzie, 2018).

The aim of this work is to investigate the degree of this proposed alignment in a case study inspired by the recent Italian court ruling on the reputational-ranking algorithm used by a food delivery platform (Keane, 2021). I rely on the legal dispute documentation to discuss the applicability, intuitiveness, and appropriateness of causal models in evaluating fairness, placing a specific focus on the causal-based fairness definition of “counterfactual fairness” (Kusner et al., 2017; Yang et al., 2020).

## Counterfactual fairness

Following Pearl (2009), and Kusner et al. (2017) we define a causal model as a triple M=(U,V,F) in which V represents the set of observed variables, U represents a set of background variables not caused by any variable in V , and F represents a set of functions $\left\{f_{1},\ldots f_{n}\right\}$ . Each function $f_{i}\in F$ corresponds to an observed variable $V_{i}\in V$ such that $v_{i}=f_{i}(pa_{i},u_{i}$ ), where $PA_{i}\subseteq V-\left\{V_{i}\right\}$ and $U_{i}\subseteq U$ . According to the structural causal model M , the value of each variable $V_{i}$ is assigned through a deterministic function $f_{i}$ of the values of the parent variables $PA_{i}$ and background variable $U_{i}$ (Pearl, 2009).

The causal diagram corresponding to the causal model is a Directed Acyclic Graph (DAG) in which every node represents a variable and directed arrows are drawn from $PA_{i}$ and $U_{i}$ to $V_{i}$ (Pearl, 2009). Assuming that the functions in F represent independent physical mechanisms, causal models are incredibly useful to obtain information on the variables under external interventions (Pearl, 2009; Pearl and Mackenzie, 2018). Under the strong assumption that all functions in F are correctly specified and that the distribution of the variables in U , P(u) , is known, it is possible to use the causal model M to calculate counterfactual quantities (Pearl, 2009). Given a probabilistic causal model 〈M,P(u)〉 , the counterfactual quantity $P(B_{A\leftarrow a}\left(U\right)=b\;|\;E=e)$ represents the conditional probability of event B=b if event A=a had happened, given that E=e actually happened (Pearl, 2009). For more technical details and more details about the terminology see (Pearl, 2009).

The concept of counterfactual fairness was introduced by Kusner et al. (2017) building on Pearl’s structural approach. Kusner et al. defined a typical prediction problem in which we have (1) a “protected” attribute A , being a variable we do not want to discriminate against (e.g., gender, sex, ethnicity, nationality, etc.), (2) a set of other non-protected predictors X , and (3) an outcome variable Y , that we are interested in predicting. If we further have the causal model (U,V,F) , where V=A∪X , the predictor $\hat{Y}$ is counterfactually fair if the following holds under any context X=x and A =a :


$$P\left({\hat{Y}}_{A\leftarrow a}(U)=y|X=x,A=a\right)=P\left({\hat{Y}}_{A\leftarrow a\prime }(U)=y|X=x,A=a\right)$$

for all values of y and for all possible values $a^{\prime }$ (Kusner et al., 2017; Loftus et al., 2018).

Where ${\hat{Y}}_{A\leftarrow a}\left(U\right)$ indicates the counterfactual variable $\hat{Y}$ when an external intervention sets the protected attribute A to the value a . This quantity is specified by Kusner et al. as ${\hat{Y}}_{A\leftarrow a}\left(U\right)$ to explicitly indicate that it corresponds to the solution of the structural equation model for $\hat{Y}$ when A is set to a , and that this counterfactual variable is actually a function of the background variables U (Kusner et al., 2017). Indeed, randomness in the counterfactual fairness definition is induced by U , the set of background variables, whose realization u describes a specific individual (Loftus et al., 2018).

One of the main advantages of counterfactual fairness is the fact that it is an individual-level fairness criterion (Mehrabi et al., 2019), which is arguably closer to the human understanding of fairness than population-level fairness criteria (Loftus et al., 2018). A predictor $\hat{Y}$ is considered counterfactually fair if A is not a cause of $\hat{Y}$ in any individual instance (Kusner et al., 2017). Or equivalently, when the distribution of $\hat{Y}$ remains identical while changing the value of A and holding constant all variables not causally affected by A (Kusner et al., 2017). This corresponds to the intuitive argument that a predictor is fair if it produces the same predictions in the counterfactual world in which all other things are equal aside from the protected attribute, which is forced to a different value (Loftus et al., 2018).

Recently, a counterfactual fairness analog has been proposed as a criterion for producing fair rankings relying on counterfactuals (Yang et al., 2020). For a score-based ranking algorithm, fairness is achieved when the counterfactual fairness condition holds for the ranking built using the score. Indeed, a ranking $\hat{\tau }$ is counterfactually fair if the following condition holds:


$$P\left(\hat{\tau }\left(S_{A\leftarrow a}\left(U\right)\right)=k\;|\;X=x,A=a\right)=P\left(\hat{\tau }\left(S_{A\leftarrow a\prime }\left(U\right)\right)=k\;|\;X=x,A=a\right)$$

for all possible values of k,x , a and $a^{\prime }\neq a$ , and with randomized tie-breaking (Yang et al., 2020). S represents the utility score used to rank the individuals and $S_{A\leftarrow a}\left(U\right)$ represents the counterfactual value of the utility score in the scenario in which A is externally fixed to a .

An interesting consequence of counterfactual fairness definition is, generally speaking, that counterfactual fairness does not hold if the rank (or the predictor) is determined by the sensitive attribute or by a consequence of the sensitive attribute (Kusner et al., 2017). In terms of DAGs, this means that an algorithm will not generally be counterfactually fair if the utility score (or the predictor) node will be a descendant (consequence) of the sensitive attribute node (Kusner and Loftus, 2020).

## Case study

On December 31st, 2020, the court of Bologna (Italy) ruled that the reputational-ranking algorithm used by a food delivery platform operating on national territory had demonstrated discriminatory behavior in violation of labor laws (Keane, 2021). Although the food delivery platform operating in Italy had already stopped using the algorithm before the final court ruling was delivered, representatives of the union considered this verdict a historic achievement in Europe (“L’algoritmo di Deliveroo è discriminatorio”: sentenza del Tribunale di Bologna, 2021). The objective of this article is neither to give a detailed description of the court proceedings nor to discuss legal aspects or responsibilities, especially since the sentence is not definitive and further appeals are possible. In this work, I used available court documents and the sentencing rationale, which I assumed to be correct, as a basis to discuss the applicability, intuitiveness, meaning and appropriateness of counterfactual fairness. Details of the sentence can be found in the original court order [downloadable here (Deliveroo, 2021)]. In the next section, I summarize the most relevant points pertaining to the legal controversy surrounding the reputational-ranking algorithm. Immediately thereafter, I translate the story described in the court documents into a simplified causal diagram as qualitative representation of my interpretation of the data generation process (Pearl, 2009; Pearl and Mackenzie, 2018; Hernan and Robins, 2019). Causal diagrams facilitate ready understanding of how changes in certain variables are propagated to others and are an easy, visual way to describe and recognize unfair mechanisms in society (Kusner et al., 2017; Makhlouf et al., 2020).

### Description

In December 2019, an appeal to the court of Bologna was filed by three Italian unions accusing an Italian food delivery platform of discriminatory behavior in how it provided access to work (Tribunale, 2020). The food delivery platform operated in the sector of home delivery and relied on a network of riders who transported food to customers. Work distribution, management, and planning of riders happened through a digital platform that had a complex system of planification of work flows (Tribunale, 2020). The company had an optional self-service booking (SSB) system that allowed riders to flexibly prearrange work sessions, organized in time slots, made available by the company based on its anticipated needs (Tribunale, 2020). Riders could access the SSB calendar every Monday through the app and book work sessions (Tribunale, 2020). They could choose the time slots and the geographic area in which they wanted to receive delivery requests during the week. However, not all riders had the same opportunities to book work sessions (Tribunale, 2020).

This system used a reputational-ranking algorithm that profiled every rider according to two indexes and established when the rider could access the SSB calendar (Tribunale, 2020). Riders were categorized into three reputational-ranking groups that could access the SSB calendar Monday at 11:00 (high rank group), at 15:00 (mid rank group), and at 17:00 (low rank group; Tribunale, 2020). Since the work sessions were limited, individuals in the highest group had better work opportunities, as they were able to potentially book a higher number and the most remunerative time slots (Tribunale, 2020). One witness who worked as a rider stated that in the high rank group, it was possible to book up to 40 work hours, while only 1 or 2 h could be booked in the low rank group (Tribunale, 2020). The indexes that ultimately determined the reputational-ranking of the rider were reliability and participation (Tribunale, 2020).

The reliability index was computed based on the number of times the rider did not join a previously booked (and not canceled) work session in the past (Tribunale, 2020). Specifically, to be considered as having “joined,” the rider needed to be in the agreed-upon geographical area and log into the app within 15 min of the beginning of the scheduled work session (Tribunale, 2020).

The participation index was computed based on the number of work sessions the rider had booked in the past, during peak demand periods, such as evenings on the weekend (Tribunale, 2020). Whether the late cancellation of a booked session (within the 24 h before the beginning of the session) actually resulted in a reduction in the reliability index was a matter of debate (Tribunale, 2020).

The unions argued that this system discriminated against riders who adhered to trade-union initiatives and that it inhibited the right to strike (Tribunale, 2020). Indeed, riders who decided to take part in such collective actions would see their indexes lowered. Involvement in such initiatives meant they would possibly recede into the reputational-ranking group, and therefore, have fewer work opportunities in the future (Tribunale, 2020). The court supported this view, arguing that adhering to a strike (or not going to work for other legitimate reasons) caused a reduction in worker’s indexes and reputational-ranking and that, consequentially, the reputational-ranking algorithm demonstrated discriminatory behavior (Tribunale, 2020). The problem was that, due to a specific design choice, the algorithm did not consider the cause for absences from work. The reputational-ranking algorithm treated absences due to futile or legitimate reasons the same way (Tribunale, 2020). According to the court, this “equality of treatment” for different situations ultimately resulted in indirect discrimination (Mehrabi et al., 2019) through the penalization of a category of riders who did not go to work for legitimate reasons (Tribunale, 2020).

### Court’s rationale

The company did not provide the court with the details on how the reputational-ranking algorithm worked (Tribunale, 2020). Therefore, obtaining a clear picture of which parameters were considered, and in which way they were processed by the algorithm, is impossible. The motivation of the court was based on the belief, supported by witness declarations and official documents, that the following causal statement was true: adherence to a strike (or not going to work for other legitimate reasons) can cause a reduction in the rider’s ranking.

**Figure 1:**
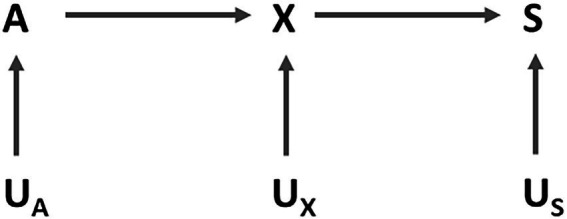
A Directed Acyclic Graph representing the causal model for the case study. A is the protected attribute (adhering to a strike), X is the variable indicating whether the rider showed up to work as scheduled or not, and S represents the score used for ranking the riders. Nodes denoted by U represent unmeasured background variables.

To translate this reasoning into a causal diagram, let A be the decision of not attending work on a specific day for a justified reason according to Italian law; this represents the protected attribute in our fairness problem. For the sake of simplicity, let us imagine that A=1 indicates that a rider adheres to a strike and A=0 indicates that a rider does not. This decision will be caused by the background variable $U_{A}$ , which summarizes the propensity of a rider to adhere to a strike. On the other hand, X will be the variable indicating whether, on that specific day, the rider showed up on time to the workplace ( X=1 ) or not ( X=0 ). Let us further define $U_{X}$ , a summary variable indicating other reasons not to show up to work. As the court clearly stated, adhering to a strike implies the material behavior of not showing up to work on time (Tribunale, 2020). For this reason, we can draw a direct arrow from the node A to the node X in the DAG depicting the causal structure of the problem (Figure 1).

We further define as S the final score, which we know depends on the participation index and the reliability index, used to rank riders. The court gathered enough evidence to be certain that not showing up to work inevitably leads to a reduction in the reliability index and maybe (depending on the day and time) in the participation index (Tribunale, 2020). Therefore, not showing up to work on time ( X ) impacts the overall final score ( S ), and we can draw an arrow between these two nodes (Figure 1). Finally, since we do not know exactly how the algorithm works, we can introduce a node $U_{S}$ which represents possible unmeasured other determinants of the final score S . The court judged the algorithm to have a discriminatory behavior because adhering to a strike causes a reduction in the score, and therefore, a lowering of the rider’s rank with a consequent disadvantage to his/her future work opportunities (Tribunale, 2020). Indeed, since S is a descendant of A (Figure 1), the distribution of the ranks determined by S will likely change if we change the value of A while holding constant all other variables not causally affected by A (therefore leaving X free to change based on A ), violating the counterfactual fairness definition.

A crucial passage in the court documentation quite clearly illustrates how close the concept of counterfactual fairness was to the court order rationale. When the company argued that as long as a rider logged into the app (even without delivering any orders), he/she would not lose points, stating that the algorithm is “blind” towards the causes of work absences, the judge dismissed this position. Indeed, joining a strike was considered incompatible with showing up to the workplace, and the act of giving advanced notice of the worker’s intent to strike would give the company the opportunity to easily replace the worker, nullifying the effects of the strike (Tribunale, 2020). This reasoning also applied to other legitimate reasons for absence, such as sickness, disability, or care of a minor, because they implied that the worker could not leave their residence to go to the specified geographic area and log-in on time (Tribunale, 2020).

This passage reinforces the idea that A (striking or other legitimate absence) is a cause of X (not showing up to the workplace on time). More importantly, the court clarifies that the contrast of interest was between the rank determined by the score ( S ) for the same context, characterized in our example by $U_{X}$ and $U_{S}$ , if A changes from 0 to 1 without holding the consequence of A ( X , showing up to work) fixed. The contrast considered by the court is precisely the counterfactual contrast of interest in the counterfactual fairness definition. The variable X, showing up to work, was not considered a so-called resolving mediator (Yang et al., 2020). Indeed, the fact that the algorithm did not discriminate against the rider in the unlikely scenario, in which he/she decides to strike but nevertheless shows up to the workplace at the scheduled time and place, was considered irrelevant. Wang et al. clearly distinguished these two levels of fairness: while the fairness definition more representative of the court’s decision is that of counterfactual fairness outlined above (also called “affirmative action” by Wang et al.), whereas the equality of treatment corresponds to Wang et al.’s definition of “equal opportunity” (a form of counterfactual fairness in which X is considered a resolving mediator (Wang et al., 2019; Yang et al., 2020)). Equality of treatment (or equality of opportunity) resulted, according to the court, in an indirect discrimination. Thus, the apparently “neutral” decision rule, blind to the reasons of the work absence, resulted in a disadvantage for a specific group of workers (Tribunale, 2020).

The definitions of direct discrimination and indirect discrimination are outlined in the court’s documentation. These definitions, especially of direct discrimination, closely mirror the counterfactual definition of fairness (Tribunale, 2020). Especially considering that a few sentences after presenting the definition, the judge cites a decree making explicit that the definition of direct discrimination refers not only to an observed, factual comparison, but also to a hypothetical one (Tribunale, 2020).

Interestingly, during the course of the trial, it became evident that if the rider experienced an accident during working hours or if the digital platform did not function properly, the reputational-ranking algorithm did not deduct points on the indexes but relied on a “simulation” to avoid penalizing workers for these specific attributes (Tribunale, 2020). This means that the company identified some causes of X as “protected” (in our DAG in Figure 1, these causes were not explicitly depicted and were part of $U_{X}$ ), and relied on complex statistical techniques to make fair decisions with respect to these attributes. According to the court, it became clear that the blindness of the reputational-ranking algorithm towards some specific causes of work absence was a deliberate design choice of the company, rather than a technical limitation (Tribunale, 2020).

## Discussion

In this article, I have shown how the definition of counterfactual fairness (Kusner et al., 2017; Yang et al., 2020) closely aligns with the human intuition of fairness expressed in a recent Italian court ruling on the reputational-ranking algorithm used by a food delivery platform.

The court’s line of reasoning was indeed oriented towards identifying the causal links between the individual components of the problem. Once the presence and direction of the cause-effect relationships were established relying on witness declaration and documentation, the court’s order emphasized a specific contrast of scenarios used to decide whether the algorithm could be considered fair. The described comparison appears to match the counterfactual contrast deemed to be relevant by the counterfactual fairness definition. This finding was strengthened by the judge’s explicit reference to a hypothetical comparison (i.e., use of counterfactual thinking) to define discrimination. The company using the algorithm, which considered “equality of opportunity” an appropriate fairness criterion when considering the protected attribute “adhere to a strike,” conceptualized fairness differently when it came to different attributes.

Generally speaking, causal models represent a powerful tool to formalize the concept of fairness. This is evidenced by the fact that, in legal texts, the definition of discrimination closely resembles an evaluation of counterfactual statements (Pearl and Mackenzie, 2018). Building an accurate causal model requires a thorough understanding of the real world processes in which the algorithm is to be applied. Nevertheless, through this example, we observe that in order to identify the absence of counterfactual fairness in certain scenarios, it appears neither necessary to have detailed information about how the prediction model is built, nor to have large datasets or detailed knowledge of all the causal mechanisms at stake. This work shows that when developing an algorithm, it is of paramount importance to consider the societal impact of its application and that causal knowledge of the context to which the algorithm is applied is crucial in order to detect and avoid discriminatory applications thereof. In light of the rapid expansion of algorithm use in business, researchers and policy makers must define rigorous and meaningful fairness metrics that allow for the detection and correction of algorithmic discriminatory behaviors in a formal and structured way.

This work has some limitations. First, I compared the counterfactual fairness definition to my interpretation of the court order. I acknowledge that my interpretation of the court rationale lies solely in my understanding of the written documentation and this may not fully mirror the exact reasoning the court used to reach its decision. However, I want to emphasize that another independent scholar who recently described the court’s rationale from a legal perspective identified the same causal connections as crucial to the court’s decision (Pietrogiovanni, 2021). Second, I took inspiration from the court order to build a simplified causal model, which clearly does not describe all the complexities of the real world. However, the nodes relevant to my line of argument, I believe, are completely depicted.

Moreover, as previously stated, I assumed the reasoning of the court to be a “correct” way to assess whether a decision-making process was fair or not. This is in line with the idea that human intuition represents the “gold standard” to evaluate fairness (Nabi and Shpitser, 2018).

Several metrics and definitions of fairness exist, some of which I have briefly reviewed in previous sections. However, there is no consensus on which metric can be used in a given scenario (Verma and Rubin, 2018; Mehrabi et al., 2019), and the use of different metrics have led to divergent results in the past (Mitchell et al., 2021).

I believe that this lack of consensus stems from the fact that is impossible to formalize a unique, universally-accepted definition of fairness. Since different preferences, social roles, material interests and cultural elements (among other factors) influence the way humans conceptualize fairness, no definition will be universally accepted by everyone in all situations at all times (Mehrabi et al., 2019).

I think the challenge and purpose of algorithmic fairness, as a research branch, is to identify different ways in which societal groups reason about fairness in different contexts and translate those reasoning into metrics. A first step in this direction is comparing examples of human reasoning about fairness with the available definitions of algorithmic fairness and select the definitions that more closely parallel the established human “gold standard” in specific applications and settings. In the case study I have presented, three unique perspectives emerge on the specific issue: (1) that of the company, (2) that of the workers/unions, and (3) that of the court (which was my focus in this article), through its interpretation of the Italian law.

It will probably not be possible to provide a general definition of algorithmic fairness, but it may be possible to explicitly formalize what different groups of individuals understand under “fairness” in different contexts. A work that goes in this direction is the study from Saxena et al. assessing general public attitudes toward three definitions of algorithmic fairness (Saxena et al., 2020).

I am hopeful that through an interdisciplinary approach, bringing together elements of law, sociology, psychology, ethics, and data science, we will be able to ascertain which fairness criteria better mimic human reasoning about fairness under certain scenarios and for certain social groups. These criteria will then be most suitable for incorporation into algorithmic development for the specific societal perspective selected.

## Data availability statement

The original contributions presented in the study are included in the article/supplementary material, further inquiries can be directed to the corresponding author.

## Author contributions

MP conceptualized and designed the work, wrote the manuscript, conceived the simplified example, created the figure, approved the final version of the manuscript, and is accountable for all aspects of the work.

## Conflict of interest

MP reports having been partially funded by a research grant from Novartis Pharma for a self-initiated research project, unrelated to this work. He reports being awarded a research grant from the Center for Stroke Research Berlin (private donations) for a self-initiated project, unrelated to this work, on causal diagrams.

## Publisher’s note

All claims expressed in this article are solely those of the authors and do not necessarily represent those of their affiliated organizations, or those of the publisher, the editors and the reviewers. Any product that may be evaluated in this article, or claim that may be made by its manufacturer, is not guaranteed or endorsed by the publisher.
